# Identifying Dental Anxiety in Children’s Drawings and correlating It with Frankl’s Behavior Rating Scale

**DOI:** 10.5005/jp-journals-10005-1401

**Published:** 2017-02-27

**Authors:** Jyoti Mathur, Amish Diwanji, Bhumi Sarvaiya, Dipal Sharma

**Affiliations:** 1 Professor and Head, Department of Pedodontics and Preventive Dentistry, Faculty of Dental Science, Dharmsinh Desai University, Nadiad, Gujarat India; 2 Senior Lecturer, Department of Pedodontics and Preventive Dentistry, Faculty of Dental Science, Dharmsinh Desai University, Nadiad, Gujarat India; 3 Former Lecturer, Department of Pedodontics and Preventive Dentistry, Faculty of Dental Science, Dharmsinh Desai University, Nadiad, Gujarat India; 4 Tutor, Department of Pedodontics and Preventive Dentistry, Faculty of Dental Science, Dharmsinh Desai University, Nadiad, Gujarat India

**Keywords:** Children’s drawings, Dental treatment anxiety, Stress markers.

## Abstract

**Aim:**

To develop a simple method to assess the level of anxiety by using children’s drawings and correlating them with Frankl’s behavior rating scale.

**Materials and methods:**

A total of 178 patients aged of 3 to 14 years were handed out two-page forms which contained three sections on coloring and drawing, along with general information, and Frankl’s behavior rating scale for the visit. The three types of drawing exercises given to the patients were geometric copy drawings, coloring a nonthreatening figure, and an empty sheet for freehand drawing.

**Results:**

Out of 178 patients, 60 showed definitely positive behavior, 73 exhibited positive behavior, 37 showed negative behavior, and 8 were definitely negative on Frankl’s behavior rating scale; 133 children had none or, 1 stress marker and 45 exhibited 2 or 3 stress markers in their drawings. Chi-square (χ^2^) analysis was done with a 2 × 2 contingency table. Observed χ^2^ value was 46.166, which at 1 degree of freedom was much greater than that at 0.995 percentile. Therefore, the result was highly significant.

**Conclusion:**

Children requiring specialized behavioral techniques can be identified by the presence of stress markers in their drawings. This nonverbal activity by itself can have an overall positive effect on the behavior displayed in the dental clinic.

**How to cite this article:**

Mathur J, Diwanji A, Sarvaiya B, Sharma D. Identifying Dental Anxiety in Children’s Drawings and correlating It with Frankl’s Behavior Rating Scale. Int J Clin Pediatr Dent 2017;10(1):24-28.

## INTRODUCTION

Dental treatment anxiety is a well-known fact. General perception in the population with regard to dental treatment is pain and discomfort. Such thought processes are bound to affect the behavior of pediatric dental patients even before the first dental appointment.^1-4^ Identification and management of child’s/patient’s behavior is an important aspect in the delivery of successful dental treatment. Many types of behavior rating scales are being used by professionals working in psychology-related fields, such as Conner’s behavior rating scale,^5^ and many types of behaviorally anchored rating scales.

One scale which is commonly used in dentistry is Frankl’s behavior rating scale,^6,7^ popular because of its ease of learning and usage. It allows a quick classification of the child patient in one of four categories: Definitely positive, positive, negative, and definitely negative.

Children learn to draw and color at an early age, and as a fact, all children are attracted toward such activities which can be used as a means of nonverbal communication by them. Children’s drawings have been used in hospital settings to assess the anxiety levels in admitted patients.^8^ Written essays^9^ and physiological parameters like changes in electrodermal activity,^10^ measurement of salivary amylase,^11^ blood pressure (BP), and pulse rate have also been used as indicators of dental anxiety in children.^12,13^ Such methods, however, themselves are capable of inducing stress due to excessive questioning and/or usage of BP Cuffs, pulse oximeter or electrocardiogram machines, etc.

This article has emerged from the department of pedi-atric dentistry, out of a quest to develop a quick nonin-vasive method to assess the levels of anxiety in the patients walking in for treatment. The aim of this study, therefore, was to find out if children’s drawings could be used as an effective tool to assess dental anxiety and to establish a channel of communication with the child patient.

## MATERIALS AND METHODS

Patients between the ages 3 and 14 years were handed out two-page forms where, in addition to general information, the type of dental treatment required was classified as invasive (requiring local anesthesia) or noninvasive (not requiring local anesthesia) was also noted. Frankl’s behavior rating scale was especially noted. Rest of the form required patients to draw and color and was divided into three parts.

**Fig. 1:**
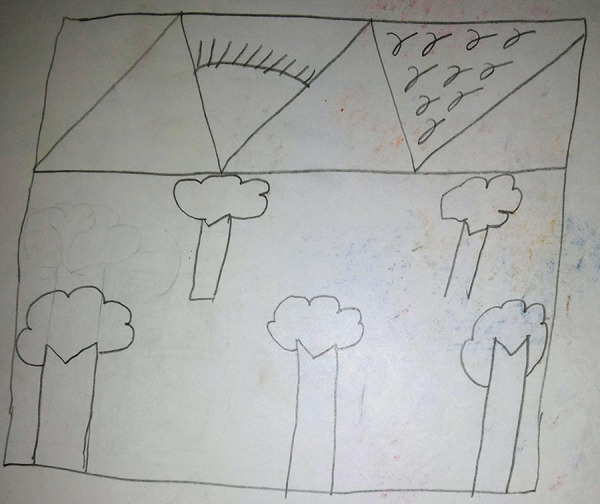
Freehand drawing of a natural scene showing mountains and trees done by an 11-year-old patient in the presence of mother showing “cookie cutter” images typical of children in an overcontrolled environment. Incidentally, this patient was “positive” on Frankl’s behavior rating scale

The first part required the children to copy geometric shapes to assess visual motor coordination at their particular age level. The second part contained a line drawing of a happy clown with ice cream (nonthreat-ening figure) which they were required to color. The third part contained an empty page where the children were asked to draw themselves doing something (also called kinetic drawings). All children with normal, age-appropriate cognitive and physical development were included in the study. Although the drawings from children with gross physical and learning defects were excluded from the present exercise, the information collected is part of another future study group with similar cohorts. While conducting the pilot study, it was observed that parents/guardians accompanying the children influenced their drawings to the level of actually dictating what to draw. One guardian went to the extent of drawing for the child! This led to inaccurate/guarded drawings by the children (e.g., Fig. 1). It was, therefore, decided to place them at a distance, but within the radius of vision of parents/guardians while completing the nonverbal tasks. The student intern in charge of the patient filled up brief explanation of the freehand drawing. It was also observed that children seated together were able to influence each other, and we found identical drawings and color usage by a group of children when seated as such. Subsequently, the children were also made to sit at a distance from each other and were encouraged to complete the tasks independently. For younger children, closer proximity with the parents was allowed, but they were specifically asked to just explain the task at hand to their wards.

## Determination of Stress Markers

After going through the responses of children in the filled form, four features were deemed as markers of stress or anxiety.

These were:

- Age discrepancy of more than or equal to 2 years in the drawings of geometric shapes/refusal to draw the geometric shapes.
- Did not complete/refused to color the figure of clown with ice cream.
- Did not draw himself/herself or any human figure.
- Refusal to draw anything.

After collection of data, it was observed that the maximum number of stress markers in a patient identified in the completed forms were 3 out of the 4 defined. Therefore, the entire study group was divided under four categories: Absence of stress markers; presence of one stress marker - one red sticker/box (■); presence of 2 stress markers - two red stickers/boxes (■■ ); and presence of three stress markers - three red stickers/boxes (■■■).

## RESULTS

Out of 178 children in the study, 75 were females and 103 were males. There were 39 children up to the age of 6 years, 84 in 7 to 10 years age group, 45 in 11 to 13 years age group, and 11 children were equal to or older than 14 years of age. Sixty children showed definitely positive behavior, 73 exhibited positive, 37 showed negative, and 8 were seen to be definitely negative on Frankl’s behavior rating scale. One hundred and thirty-three children showed none or 1 stress marker, and 45 showed 2 or 3 stress markers in their drawings. The number of females displaying Frankl’s negative and definitely negative behavior (collectively called negative behaviors) was 14 out of 75, i.e., 18.66%, and the number of males with negative behaviors was 30 out of 103, i.e., 29.12%. The difference in the proportion of males and females displaying negative behaviors was 10.46, and the standard error of proportions between them was ≈6. Therefore, as the difference was about 1.743 times the standard error which is lesser than 1.96, the gender difference for the negative behaviors was nonsignificant, though being marginally high in males.

About 41.02% of children up to the age of 6 years were behaviorally negative. This percentage was 21.68% and 22.22% for 7 to 10 years and 11 to 13 years age groups respectively. Only 1 child out of 11, i.e., 9.1% of older children, displayed any negative behavior.

A null hypothesis was formulated according to which it was assumed that no correlation exists between Frankl’s behavior rating scale and stress markers.

**Table 1:** **Table 1:** Chi-square analyses for the study

				*None or 1 stress markers*												*2 or 3 stress markers*													
*Sl.* *no.*		*Frankl’s* *scale*		*O*		*E*		*O-E*		*(O-E)* *-0.5*		*[(O-E)* *-0.5]^2^*		*[(O-E)* *-0.5]^2^/E*		*O*		*E*		*O-E*		*(O-E)* *-0.5*		*[(O-E)* *-0.5]^2^*		*[(O-E)* *-0.5]^2^/E*		*n*	
1		Def. position and positive		117		99.376		17.624		17.124		293.231		2.950		16		33.623		–17.623		17.123		293.197		8.720		133	
2		Def. negative and negative		16		33.623		–7.623		17.123		293.197		8.720		29		11.376		17.624		17.124		293.231		25.776		45	
n				133												45												178	

χ^2^ = 46.166; At degree of freedom (df) = 1, obtained χ^2^ value is much greater than that at 0.995 percentile; therefore, highly significant with probability of 0.005 occurrence by chance.

Therefore, we reject the null hypothesis.

O: Observed value; E: Expected value; n: Total number; Chi-square statistic (χ^2^) = Σ (observed value-expected value)^2^/expected value; Yates correction = 0.5 subtracted from the observed difference of the observed and expected value for cell frequency less than 5. 2 × 2 table = data consisting of two rows and two columns.

Degree of freedom (df) = (number of rows 1) × (number of columns 1). Therefore, df in a standard 2 × 2 table is 1.

*If the derived (χ^2^) value is greater than that predicted in the χ^2^ distribution table at the particular df for the required percentile, the result is considered significant or highly significant (standard χ^2^ distribution tables are available freely for reference).

A 2 × 2 contingency table was prepared with rows depicting (1) definitely positive and positive behavior and (2) negative and definitely negative behavior which were matched against columns assessing (a) none or 1 stress marker and (b) presence of 2 or 3 stress markers (Table 1).

Even with using Yates correction (Table 1), Chi-square (χ^2^) value observed was 46.166 which at 1 degree of freedom (Table 1) was much greater than that at 0.995 percentile. Therefore, the null hypothesis was rejected and the result was highly significant (Table 1) with the probability of the result occurring by chance lesser than 1 - 0.995 = 0.005.

## DISCUSSION

Information from the clinical interview/history is derived from two major sources: Verbal and nonverbal. Verbal information refers to the data the patients tell us about themselves; nonverbal communication refers to observations we make about what is not said, such as use of facial expressions, posture of a patient, movement of extremities, and quality and tone of speech.^14^

The first nonverbal task given to the children in our study was copy drawing of geometric figures. There are many such tests available, such as Bender-Gestalt test and Beery Bucktenica visual motor integration test, which are meant to assess the visual motor integration skills in children and adults. They also provide useful information for educational, psychological, and neuropsychological assessment. The one used in our study was based on screening test for visual motor integration described in the textbook Encounters with Children, Pediatric Behavior and development (4th edition). It gives the age by which children are able to draw the given geometric figures.^15^ Any discrepancy in the ability to draw may point out a deficiency in cooperation levels in a stressful condition. The cutoff level for discrepancy decided by us was ±2 years between the drawn figures and the accepted norms for age.

The second nonverbal task involved coloring a friendly/nonthreatening image of a clown with an ice cream based on the finding that children are able to alter their use of color during picture completion tasks in response to topic characterizations and even very young children are able to use colors symbolically.^16^ Those children in our study who did not complete coloring the picture or refused to color the image were assumed to be under dental stress, and this information was used as a stress marker.

The third and last part of the nonverbal history sheet contained an empty page where children could draw using a pencil. This part was based on various projective tests the most famous and useful being Goodenough-Harris Draw-a-Person test (1926). It is typically used with children where the subjects are asked to draw a picture of themselves, family, or the people surrounding them. These are analyzed on a number of dimensions, such as facial expressions, number of facial features drawn, proportion of body parts. Such projective tests have been critically analyzed in the past citing lack of reliability and validity, the weak and inconsistent research base, their susceptibility to contextual and situational factors (e.g., mood of examiner or biased use of language and subjectivity in scoring and interpretation). Therefore, in our study, we have refrained from analyzing individual drawings. Instead, we have used the information of patients’ refusal to draw a human figure (self or someone else) or refusal to draw any figure as one of the indicators of dental stress.

**Figs 2A to C:**
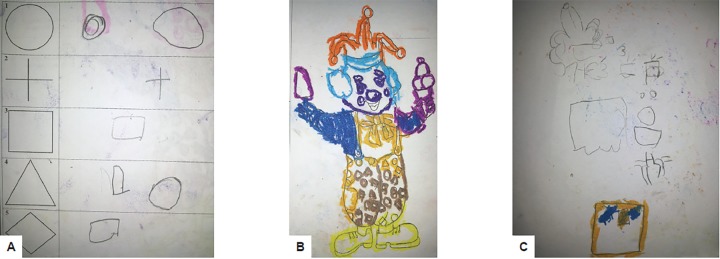
(A) Geometric copy drawings done by a 9-year-old female patient displaying Frankl’s negative behavior during dental treatment. These are at the level of copy drawing expected from 41/- to 51/-year age group; (B) coloring task left incomplete by the same patient as in 2A; and (C) no clarity of thought, disjointed figures, and no resemblance to any object in freehand pencil drawings done by the same patient who has drawn in 2A and 2B

**Figs 3A to C:**
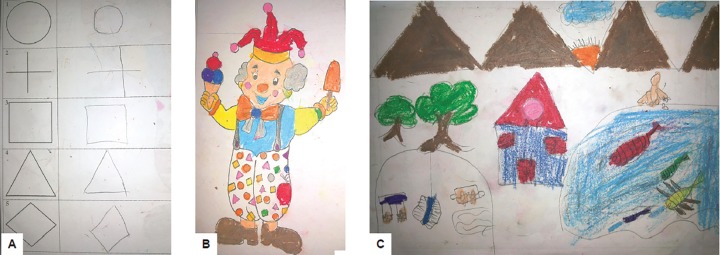
(A) Geometric copy drawings done by a 9-year-old female patient displaying Frankl’s positive behavior during dental treatment, showing clear, bold, age-appropriate pattern; (B) neatly completed coloring task using multiple colors by the same patients as in 3A; and (C) clarity of thought and proportion in detailing with multiple human figures drawn by the same patient as in 3A and 3B

Figures 2A to C are images drawn by a 9-year-old Frankl’s behaviorally negative female patient, which are in obvious contrast to the ones drawn by another 9-year-old female patient with Frankl’s positive behavior (Figs 3A to C).

Other researchers in the past too have found children’s drawings helpful in measuring anxiety during dental treatment.^17,18^

## CONCLUSION

Nonverbal communication with children is as important as the spoken word. By understanding children’s drawings, we get a step closer to their perception of the surroundings. In the recent past, however, this important channel of communication with our child patients has been neglected/overlooked. In our study, by the use of simple 2 × 2 contingency table for chi-square analysis, we were able to prove that there exists a definite correlation between absence/presence of designated stress markers in drawings and Frankl’s behavior rating scale which can be used to identify children requiring specialized behavioral techniques. Even otherwise, friendly activities, such as drawing and coloring help in creating a child-friendly atmosphere. These can act as “ice-breakers” between the pediatric dentist and the patients/parents that can play an important role in successful delivery of treatment. A marginally high number of male children displaying negative behaviors may be a cultural peculiarity. This article is important to pediatric dentists as:

- It attempts at increasing our understanding of child behavior, specially pertaining to dental treatment through nonthreatening activity like drawing and coloring.
- The nonverbal activity creates a novel and child/ guardian-friendly methodology to identify dental stress in individual patients even before the commencement of dental treatment.
- Appropriate behavior modification techniques can be applied for the patients thus identified.
